# Identification and validation of neutrophil-related biomarkers in acute-on-chronic liver failure

**DOI:** 10.3389/fimmu.2025.1477342

**Published:** 2025-02-25

**Authors:** Wei Lin, Yongping Chen, Mingqin Lu, Cheng Peng, Xiang Chen, Xiaoqin Liu, Yunyun Wang

**Affiliations:** ^1^ Department of Orthopedics, The Third Affiliated Hospital of Wenzhou Medical University, Wenzhou, Zhejiang, China; ^2^ Department of Infectious Diseases, The First Affiliated Hospital of Wenzhou Medical University, Wenzhou, Zhejiang, China; ^3^ Zhejiang Provincial Key Laboratory for Accurate Diagnosis and Treatment of Chronic Liver Diseases, The First Affiliated Hospital of Wenzhou Medical University, Wenzhou, Zhejiang, China

**Keywords:** ACLF, WGCNA, neutrophil, biomarker genes, CIBERSORT

## Abstract

Dysfunction of peripheral blood neutrophils occurs in acute-on-chronic liver failure (ACLF). However, the molecular mechanisms of neutrophils involved in the pathophysiology of the ACLF remains poorly understood. Data downloaded from the GEO database (GSE142255) was used to identify both ACLF and neutrophil-related genes with the help of the limma package and Weighted Gene Co-Expression Network Analysis (WGCNA) algorithms. The analysis identified 288 ACLF-related differentially expressed genes (DEGs) in the circulating blood cells. Among these, three genes were found to be related to neutrophils and were identified as diagnostic genes, exhibiting high diagnostic efficacy as evidenced by an area under the curve (AUC) value of 1. Among these, matrix metallopeptidase-9 (MMP9) and S100 calcium binding protein A12 (S100A12) were upregulated, whereas C-C chemokine ligand 5 (CCL5) was downregulated in circulating immune cells from patients with ACLF compared to those from healthy controls. These findings were corroborated using an additional GEO dataset, GSE156382. The expression levels of the three key genes demonstrated a correlation with both ferroptosis and cuprotosis. Among the three diagnostic genes, only MMP9 was validated as differentially expressed through both quantitative real-time PCR (qRT-PCR) and western blot. Moreover, a significant elevation in plasma MMP9 levels was observed in patients with ACLF compared to those with chronic hepatitis B (CHB) and acute decompensated cirrhosis (AD). Notably, ACLF patients exhibiting elevated MMP9 levels (>175.8 ng/mL) experienced higher short-term mortality rates within both 30 and 90 days (p<0.001). In addition, a total of 21 drugs targeting the three diagnostic genes were identified from the Drug Bank database. Finally, the Kinase-TF-mRNA-miRNA network was constructed utilizing Cytoscape software. This study represents the initial application of WGCNA algorithms to identify novel biomarkers related to neutrophils in ACLF. Our findings offer new perspectives on the role of neutrophil in the pathogenesis of ACLF. However, additional research is required to substantiate the effects of these key genes and therapeutic agents on ACLF.

## Introduction

ACLF occurs when liver function deteriorates suddenly among patients with chronic liver disease, leading to multiple organ system failures and high short-term mortality (50-90%) (1). Alcoholism, bacterial infections, and chronic viral hepatitis relapse are the predominant precipitating factors in approximately 50% of patients diagnosed with ACLF (2, 3). In the absence of triggering factors, they are likely related to intestinal translocation of bacterial products (4). Previous studies have shown that systemic inflammation and immune dysfunction are considered the major factors leading to extensive tissue damage and organ failure in patients with acute decompensation (AD) that progresses to ACLF (4, 5).

Neutrophils are the major component of the human innate immune system. The granules they store contain effective antimicrobial agents. They not only play an important role in antimicrobials, but also damage host tissues in certain conditions (6). Previous studies have revealed that the neutrophil lymphocyte ratio (NLR) serves as a predictive factor for disease progression and mortality in patients with hepatitis B related acute-on-chronic liver failure (HB-ACLF) (7, 8). Studies also have identified distinct phenotypes of neutrophil in ACLF, such as elevated expression of CXCR1/2, impaired phagocytosis, varying expression of Toll like receptors (TLRs), and enhanced production of neutrophil extracellular traps (NETs) (6, 9–11). Our previous study had shown that neutrophils isolated from patients with HBV-relate ACLF (HB-ACLF) exhibited an increased production of ROS in response to bacterial stimulation compared to those from individuals with hepatitis B (5). Furthermore, it is reported that the distinct state of neutrophils is correlated with the prognosis of ACLF (11). The ability of neutrophils from ACLF patients to produce NETs is increased, which was more prominently observed in patients with unfavorable outcomes (11). Among patients with HB-ACLF, pyroptosis is the most common type of cell death in the liver. Neutrophils selectively accumulate in pyroptotic liver, aggravating inflammation by producing pathogenic NETs in ACLF (12). However, the underlying molecular mechanism remains unclear. Therefore, exploring new neutrophil-related biomarkers to identify the molecular features of neutrophils in ACLF is of great significance. Here, Based on bio-informatics methods, we analyzed the role of neutrophil-related genes in the diagnosis and prognosis of ACLF.

## Method

### Screening of microarray data

We downloaded GSE142255 and GSE156382 from the Gene Expression Omnibus (http://www.ncbi.nlm.nih.gov/geo/). The GSE142255 dataset, which was extracted on the GPL17586 platform (Affymetrix Human Transcriptome Array 2.0), comprises transcriptome analysis of whole blood samples from 17 ACLF patients, 7 acute decompensated cirrhosis patients, 7 stable cirrhosis, 5 fibrosis patients and 7 healthy individuals (6). Meanwhile the dataset GSE156382, which was derived on the GPL20844 platform (Agilent-072363 SurePrint G3 Human GE v3 8x60K Microarray 039494), includes circulating neutrophil samples from 6 chronic liver disease, 12 ACLF samples and 5 healthy samples (13). Further analysis were all performed on the data from ACLF patients and healthy controls. GSE142255 was used as the training set, while GSE156382 was utilized as verifying datasets.

### Identification of DEGs related to ACLF and neutrophil

The DEGs between patients and normal controls in the GSE142255 dataset were screened by R software “limma” package. DEG identification threshold values were set at | logFC) |>1 and P < 0.05. Volcano maps and heatmaps were plotted using the R software packages ggplots and pheatmap. Through published literature, 137 neutrophil-related genes (Ne-related genes, NRGs) were attained to screen for neutrophil-related DEGs (14). These genes were selected based on their roles in neutrophil function, infection and immune response.

To specifically investigate the role of neutrophil in ACLF, we focused on genes that were both DEGs in ACLF patients and related to neutrophil. WGCNA was performed on the samples and data in the training dataset using the R package WGCNA to identify key modules, select an appropriate soft threshold (β), and adjust the scale-free topology fitting index to exceed 0.9. Based on Pearson correlation analysis, the module most relevant to disease traits was selected as the key module, and genes from this key module were extracted as ModelGenes.

To obtain differentially expressed NRGs related to ACLF (ACLF-NRGs), we took the intersection of DEGs, NRG, and module genes (ModelGenes) related to ACLF. We aimed to pinpoint the most relevant genes that could serve as specific markers or therapeutic targets related to neutrophil function in the context of ACLF. This targeted approach allows us to highlight the molecular mechanisms and pathways directly involved in neutrophil function, which might be overlooked if all DEGs were analyzed collectively. Network module analysis is vulnerable to the impact of outlier samples; therefore, we employed a hierarchical clustering tree to identify and eliminate these outliers from the dataset.

### Localization, enrichment and protein–protein interaction analysis of the ACLF-NRGs

ACLF-NRGs were mapped onto human chromosomes by using the RCircos package of R language. We performed Gene Ontology (GO) and Kyoto Encyclopedia of Genes and Genomes (KEGG) functional enrichment analysis on ACLF-NRGs using the ClusterProfiler package, with p.adj<0.05 and count ≥ 2 as the threshold for screening. The Cytoscape STRING database was used to analyze the protein-protein interaction (PPI) networks of ACLF-NRGs.

### Identification of diagnostic biomarkers

Random Forest (RF) analysis was performed on ACLF-NRGs by using the random Forest package. The optimal number of classification trees and gene importance ranking were obtained. The selection criteria for this analysis was to include genes with mean decrease accuracy that is not zero. To eliminate the recursive features of the obtained differential genes, the SVM algorithm in R package e1071was employed. The RF method was used to sort and calculate the above feature genes, determining the significance and ranking of each gene, along with the error rate and accuracy for each iterative combination. The combination yielding the lowest error rate was identified as the optimal configuration, and the corresponding gene was designated as the feature gene. The intersection of genes obtained by RF and SVM-RFE algorithms was used to obtain feature genes.

Lasso analysis was conducted on feature genes employing the glmnet package to obtain error maps, gene co-efficient maps for cross validation and diagnostic genes. The optimal model parameter λ=0.008889174 was selected using 10 fold cross validation (the λ corresponding to the smallest error mean). These diagnostic genes were used to construct diagnostic models in the ACLF and Control groups to evaluate the diagnostic ability of those key genes for ACLF. The Receiver Operating Characteristic (ROC) analysis was conducted on this model to assess its judgment accuracy using training set GSE142255. The lasso diagnostic model constructed by diagnostic genes was validated in datasets GSE156382.

### IPA and GSEA of diagnostic genes

To better understand the molecular mechanisms of diagnostic genes acting on ACLF, signaling pathways affected significantly by diagnostic genes were identified using Ingenuity Pathway Analysis (IPA). We performed enrichment analysis on all differentially expressed genes as input, displayed the pathways in which the diagnostic genes were involved, and observed whether the enriched pathways contained disease-related pathways. “c2. cp. kegg. v11.0. symbols” was downloaded as the internal reference gene set from MSigDB database. Then Gene Set Enrichment Analysis (GSEA) was conducted on the diagnostic genes.

### Ferroptosis and cuprotosis related scores

Through published literature, ferroptosis-related genes (FRGs) and cuprotosis-related genes (Cuprotosis) were obtained. ssGSEA was used to analyze the ferroptosis ssGSEA score (FRGs Score) and cuprotosis ssGSEA score (Cuprotosis Score) of the ferroptosis-related gene set and cuprotosis-related gene set on GSE142255 samples.

### Patients

Enzyme-Linked Immunosorbent Assay were conducted on 116 patients recruited as outpatients or inpatients at the First Affiliated Hospital of Zhejiang University between August 2015 and September 2017. These patients were divided into four groups: 82 chronic hepatitis B (CHB) patients with ACLF (HB-ACLF), 8 CHB patients with acute decompensated cirrhosis (HB-AD), 11 age-/sex-matched CHB and 15 age-/sex-matched healthy subjects. RT-qPCR and western blot analysis was carried out on white blood cells from a different cohort of 54 subjects, who were recruited from inpatients of the First Affiliated Hospital of Wenzhou Medical University between July 2023 and December 2024. The Asian-Pacific Association for the Study of the Liver (APASL) defines ACLF as follows: “acute hepatic insult manifesting as jaundice (≥5 mg/dL) and coagulopathy, complicated within 4 weeks by ascites and/or encephalopathy in a patient with previously diagnosed or undiagnosed chronic liver disease.” (15) The AD patients presented with cirrhosis acutely complicated with hepatic encephalopathy, ascites and upper gastrointestinal bleeding. The diagnosis of cirrhosis was based on liver biopsy, radiological evidence, or clinical manifestation of liver decompensation. CHB is diagnosed when serum HBsAg is positive for at least six months, coupled with cirrhosis or liver fibrosis or long-term liver inflammation confirmed by histology or imaging or laboratorial or clinical evidence; Control subjects were 18 age-/sex-matched healthy individuals. We excluded patients who were infected with HIV, younger than 18 years old, pregnant, using immunotherapy, had a history of autoimmune diseases, cancer or complicated with serious diseases of other major organs. Each subject provided written consent. If participants were unable to give informed consent, their next of kin provided written consent. The Helsinki Declaration was followed in the conduct of this study. Approval for this study was obtained from the Ethic Committee of the First Affiliated Hospital of Zhejiang University and the First Affiliated Hospital of Wenzhou Medical University. A summary of the baseline characteristics of the patients can be found in the **Supplementary Tables S1** and **S2**.

### RT−qPCR analysis

Human blood samples were lysed with Red Blood Cell Lysis Buffer to obtain white blood cells. RNA extraction was conducted in accordance with the protocol provided by the TRIzol reagent manufacturer (Takara, Dalian, China). Then, the RNA was reverse transcribed into cDNA using the Primescript reverse transcription reagent kit for qPCR (Takara, Dalian, China). Finally, reverse transcription quantitative polymerase chain reaction (RT-qPCR) experiments were performed utilizing the SYBR Green Premix Pro Taq HS qPCR Kit (Takara, Dalian, China). The primers for MMP9, S100A12, CCL5 and beta-actin were offered by Meigu Biotechnology Co., Ltd. (Wenzhou, China).

### Western blot

We conducted Western blot (WB) analysis as previously described (16). Briefly, 20 μg of protein from the human blood white blood cells was subjected to SDS-PAGE using a 12% gel. After trans-membrane, the membranes were blocked with rapid blocking buffer for ten minutes at room temperature. Subsequently, the membranes were incubated overnight at 4°C with primary antibodies targeting MMP9, S100A12 and β-actin followed by incubation with secondary antibodies. At last, enhanced chemiluminescence were used to detect the protein levels. All the antibodies were purchased from Proteintech, USA.

### Enzyme-linked immunosorbent assay

After enrolling in the study, whole blood samples were collected from participants. As soon as plasma was collected after centrifugation, it was stored at -80°C immediately. In accordance with the manufacturer’s instructions, enzyme-linked immunosorbent assays from R&D and biolegend were used to determine MMP9 and CCL5 levels in plasma samples (10μL) respectively.

### Drug prediction and construction of kinase-TF-mRNA-miRNA network

Small molecule drugs for diagnostic genes were predicted using the Drug Bank database. The NetworkAnalyst database was searched for transcription factors (TFs) that regulate diagnostic genes (https://www.networkanalyst.ca/). Then the upstream kinases of the identified TFs were predicted by X2Kgui, and miRNAs associated with the diagnostic genes were predicted by using miRDB database. At last, the Kinase-TF-mRNA-miRNA network was constructed by Cytoscape software.

### Statistical analysis

GraphPad Prism 6.0 or IBM SPSS statistics 20 were used to generate and draw the statistical analysis and charts. Image J software was employed to determine the gray value of the WB. There are four ways to express data: means ± standard deviation (SD), means ± standard error of the mean (SEM), number (percentage) and median (range). For the comparison of two independent groups, Mann-Whitney U tests were used. The correlation between FRGs Score or Cuprotosis Score and the expression of the diagnostic genes were evaluated by Pearson correlation analysis. Kaplan Meier survival curves were also developed to evaluated whether plasma MMP9 levels were highly correlated with mortality. Receiver operating characteristic curves (ROC curve) were employed to calculate diagnostic capability. In all statistical tests, p less than 0.05 was considered statistically significant.

## Results

### Data processing strategy and data acquisition

**Figure 1:**
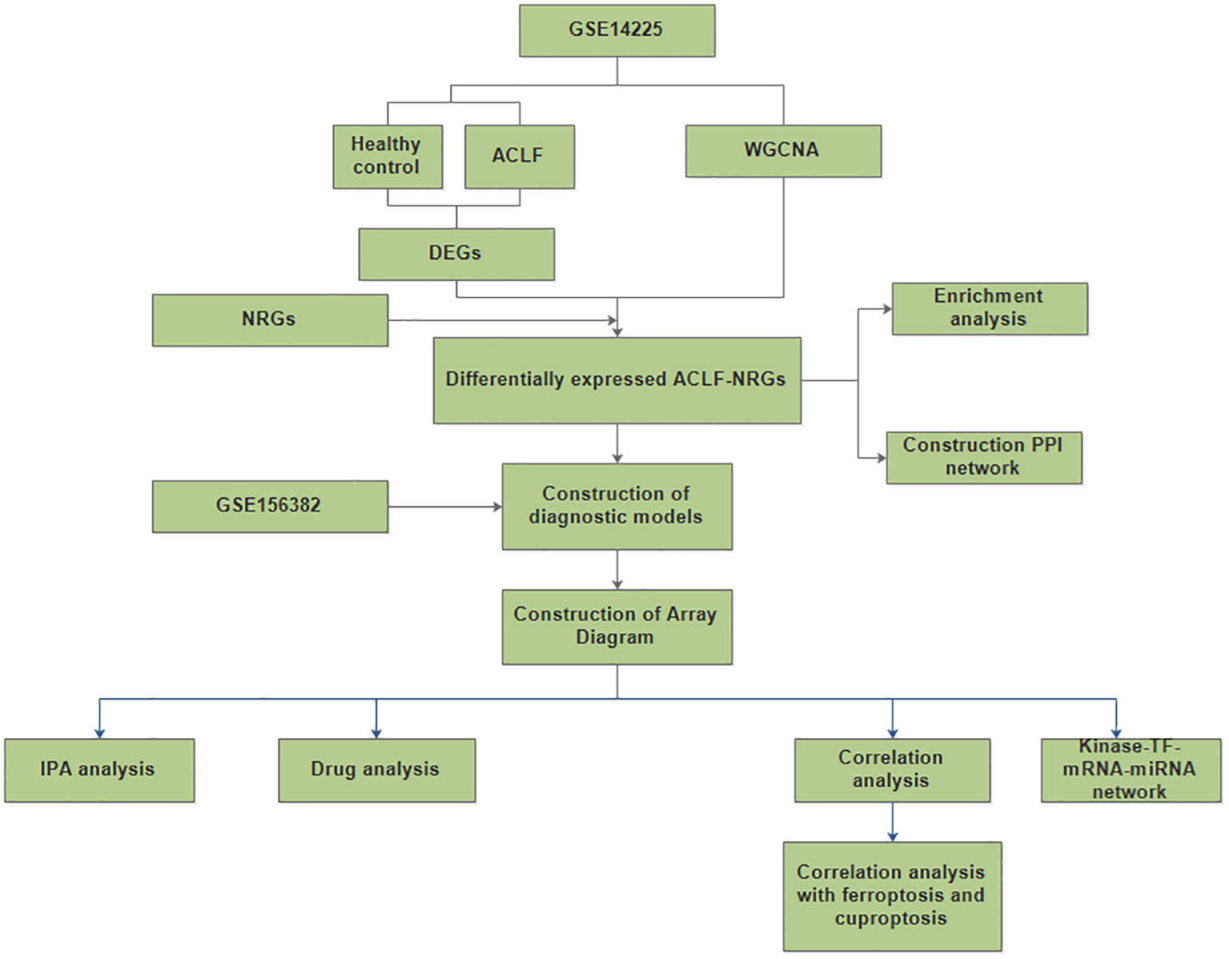
A flow for identifying novel neutrophil-related biomarkers and predicting therapeutic drugs in ACLF based on GEO data. (DEGs, differentially expressed genes; NRGs, neutrophil-related genes; ACLF-NRGs, NRGs associated with ACLF).

Study protocol was shown in **Figure 1**. The mRNA expression profiles in the GSE142255 and GSE156382 datasets were obtained from the GEO database. Samples in the GSE142255 dataset are whole blood samples, including 7 healthy samples and 17 ACLF samples (6); The samples in the GSE156382 dataset are neutrophil samples from blood, including 5 healthy samples and 12 ACLF samples (13); Through the published literature, 137 NRGs were identified (14). Both iron death and copper death-related genes were obtained from a comprehensive literature review, among which 268 ferroptosis-related genes were obtained (17). Additionally, ten cuproptosis-related genes were identified, namely LIAS, FDX1, CDKN2A, DLD, DLAT, LIPT1, GLS, MTF1,PDHB and PDHA1 (18).

### Differential analysis

**Figure 2:**
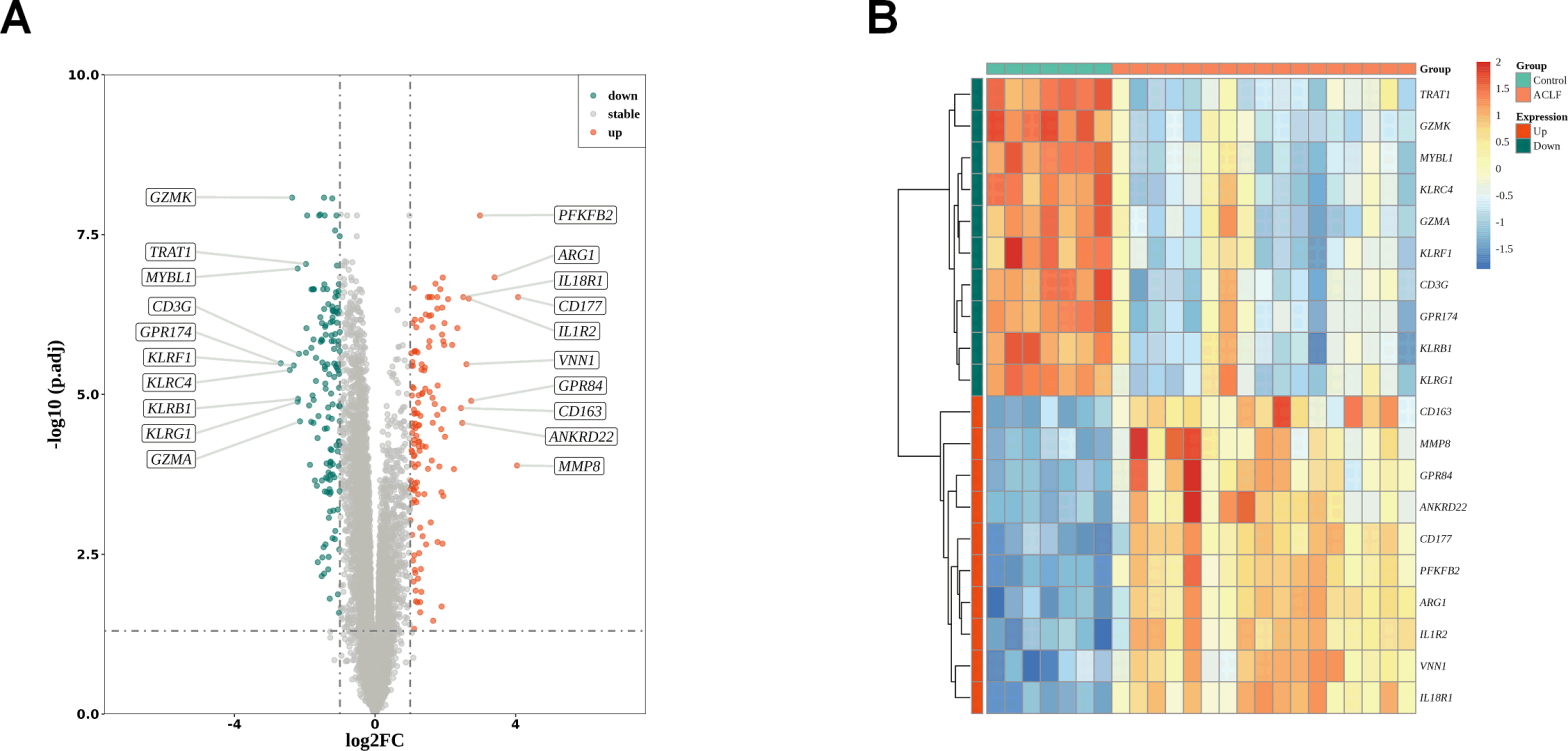
DEGs identification. **(A)** The volcano plots of DEGs between healthy control and ACLF. Green dots denote down-regulated genes, red dots signifies up-regulated genes and gray dots corresponds to genes with no significant differential expression; |log (fold change)|>1 and adjusted p <0.05. **(B)** Heat map indicating the DEGs in the healthy control and ACLF samples. The color blue signifies significantly down-regulated genes, red denotes significantly up-regulated genes, and yellow corresponds to genes with no significant differential expression.

We analyzed the differentially expressed genes between ACLF and the healthy control (HC) samples in the GSE142255 dataset using the R software limma package. To identify novel diagnostic biomarkers associated with ACLF, a total of 288 DEGs were screened. DEGs that had log fold changes greater than 1 and p-values below 0.05 after adjusting for false discovery rate (FDR) were selected. Among these DEGs, 150 genes were found to be up-regulated (**Supplementary Table S3**), while 138 genes were identified to be down-regulated (**Supplementary Table S4**). The distribution of these 288 DEGs is illustrated in the accompanying volcano plots and heat maps and 10 top upregulated and downregulated genes were selected for display (**Figures 2A, B**).

### Co-expression network construction and identification of neutrophil key modules by weighted gene co-expression network analysis

**Figure 3:**
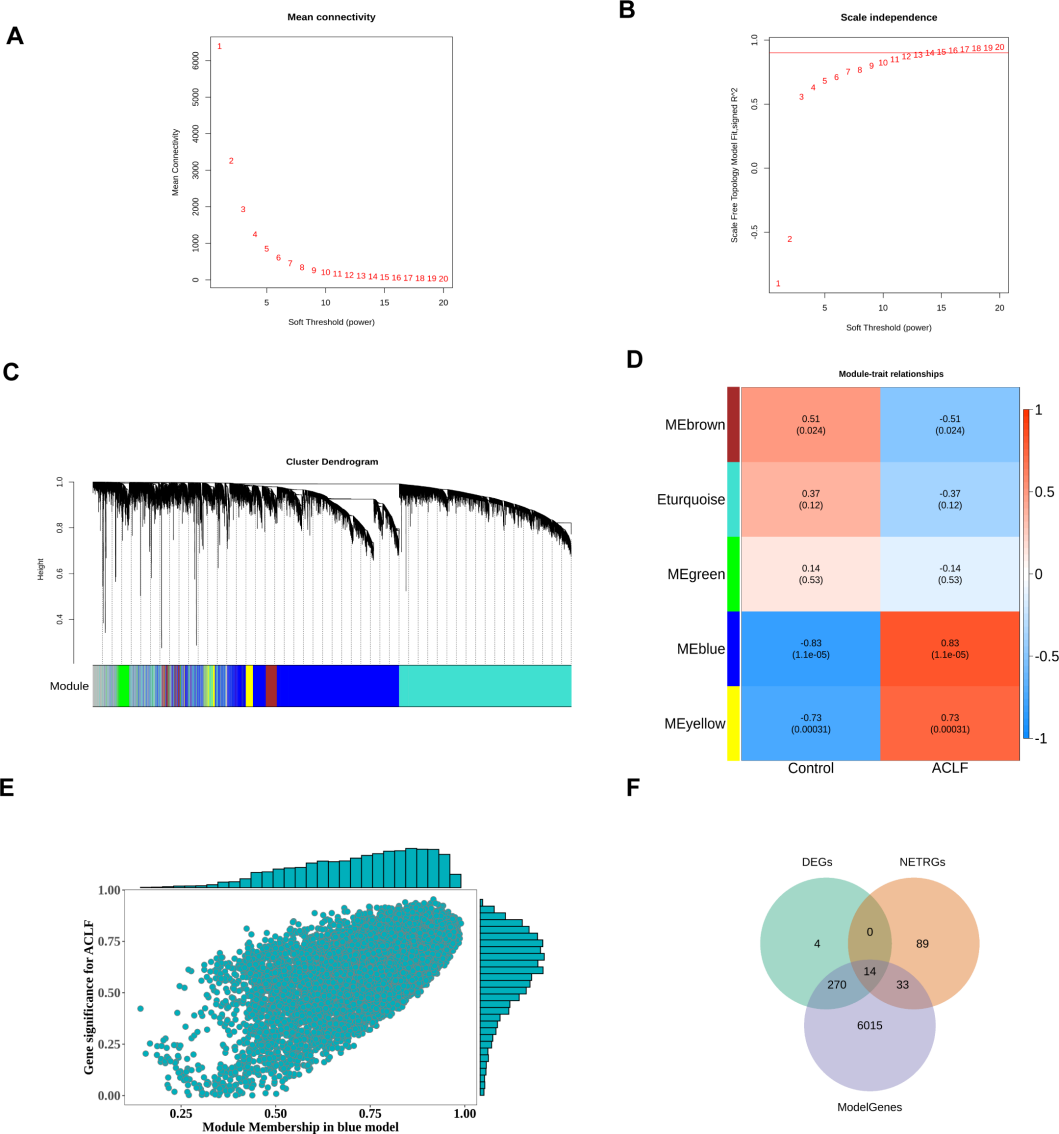
Formation of hierarchical clusters based on the soft threshold power (β). **(A)** Determination of the mean connectivity corresponding to the soft threshold power range of 1 to 20. **(B)** Evaluation of the soft threshold power’s scale independence index ranging from 1 to 20 (β= 14). **(C)** Identification and establishment of gene co-expression modules that were displayed in distinct colors in hierarchical clustering. **(D)** The correlation between gene modules and disease traits. The rows represented consensus modules, and the columns indicated group information. In every module, the correlation coefficients, along with p values, indicated the correlation between the corresponding module and group (blue indicated a negative correlation while red represented a positive correlation). **(E)** Scatter plot of the correlation between gene modules and blue modules. **(F)** Differentially expressed NRGs associated with ACLF (ACLF-NRGs).

To identify key modules, the R package WGCNA was utilized to conduct weighted correlation network analysis on the samples and data within the training set. The network module analysis is susceptible to the influence of outlier samples. We first constructed a hierarchical clustering tree for the sample (N=24), as shown in (**Supplementary Figure 1A**). The results showed the presence of outlier samples, and after removing two outlier samples (**Supplementary Figure 1B**), there were no obvious outlier samples. Therefore, the filtered samples were used for subsequent analysis. A scale-independent topological network was established, characterized by a soft-thresholding power of nine and a scale-free R² value of 0.9, along with a mean connectivity network (**Figures 3A, B**). We segmented the dendrogram at pertinent transition points using dynamic hybrid cutting to construct a hierarchical clustering tree. The leaves of the trees represent individual genes, while the branches of the dendrogram represent clusters of genes with similar expression profiles. Branches containing genes with analogous expression patterns were designated as gene modules. In total, six gene modules were produced through the amalgamation of analogous modules (**Figure 3C**). Among these six modules, the gray module is meaningless. As shown in **Figure 3D**, the blue module had the highest and significant correlation with disease phenotype (Cor=-0.8261517 p value=2.17e-06). Therefore, the 6332 genes included in this module would be marked as ModuleGenes and used for subsequent analysis. The correlation between gene modules and blue modules was shown in **Figure 3E**.

To obtain differentially expressed NRGs related to ACLF (ACLF-NRGs), an intersection of DEGs, neutrophil-related genes (NRGs), and module genes (ModelGenes) associated to ACLF was identified. As shown in **Figure 3F**, a total of 14 intersection genes (ACLF-NRGs) were obtained. Details of the 14 ACLF-NRGs were shown in the **Supplementary Materials** (**Supplementary Table S5**).

### Localization, enrichment and protein–protein interaction analysis of the ACLF-NRGs

**Figure 4:**
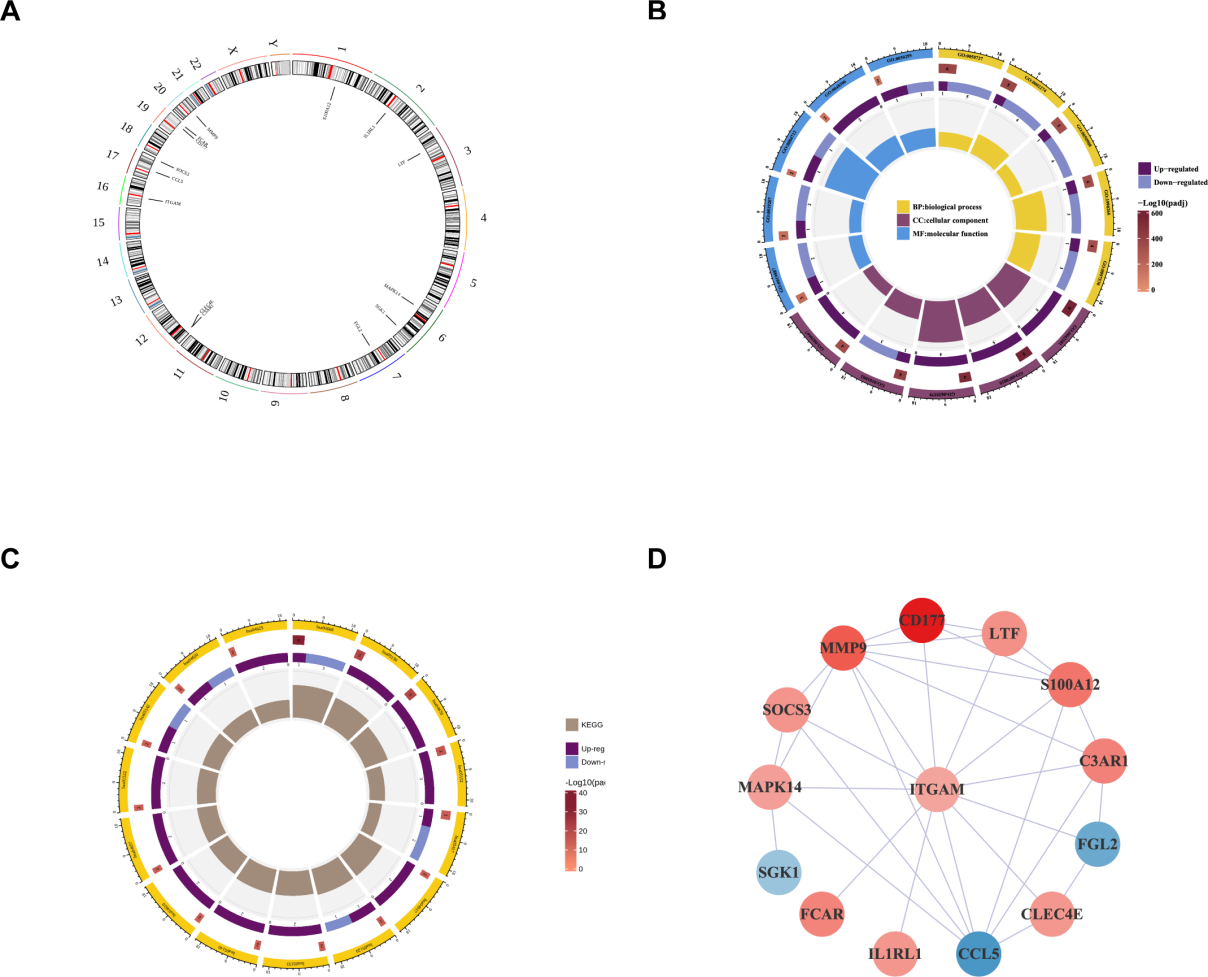
Enrichment analysis and protein-protein interaction construction of intersecting genes. **(A)** Localization of Intersecting genes on human chromosomes. The circle represents human chromosomes and displayed the location information of 14 ACLF-NRGs. **(B)** GO analysis of intersecting genes. The circles from the outside to the inside in this diagram represent: the first layer, the ID of the GO function, displayed BP, CC, and MF functions respectively; In the second layer, the color depth indicated the significance level, while the length, width, and number indicated the number of genes enriched in the function; The third layer indicated the number of up-regulated genes enriched in this function, and color was used to distinguish the genes up-regulated or down-regulated; The innermost color block, where the color indicated different functions and the size indicated the RichFactor of the pathway (The definition of RichFactor is a ratio of the number of transcripts associated with a specific Gene Ontology (GO) entry among differentially expressed transcripts to the total number of transcripts associated with that GO entry across all annotated transcripts. A higher RichFactor indicates a greater degree of enrichment). **(C)** Enrichment of intersecting genes in the KEGG pathway. **(D)** PPI network of ACLF-NRGs.

14 intersecting genes (ACLF-NRGs) were mapped onto human chromosomes by using the RCircos package in the R programming language (**Figure 4A**). As illustrated in **Figure 4B**, Gene Ontology (GO) analysis identified and examined 13 cellular components (CC), 118 biological processes (BP), and 6 molecular functions (MF) associated with these intersecting genes. The top five enrichment terminologies pertaining to BP, CC, and MF are presented in **Supplementary Table S6**. The three most significantly enriched biological process (BP) terms were related to the immune response: neutrophil degranulation, regulation of neutrophil activation, and neutrophil activation involved in the immune response. Correspondingly, the top three highly enriched cellular component (CC) terms identified through Gene Ontology (GO) analysis were specific granule, tertiary granule membrane, and tertiary granule lumen. Concurrently, the most enriched molecular function (MF) terms were protein serine/threonine/tyrosine kinase activity, calcium-dependent protein binding, and protein kinase activator activity. Comprehensive details of the Gene Ontology (GO) analysis reports can be found in the **Supplementary Materials** (**Supplementary Table S7**).

A Kyoto Encyclopedia of Genes and Genomes (KEGG) pathway analysis of the intersecting genes identified 15 immune-associated pathways, encompassing the TNF signaling pathway, Staphylococcus aureus infection, prolactin signaling pathway, epithelial cell signaling in Helicobacter pylori infection, and leukocyte transendothelial migration (**Figure 4C**). Comprehensive details of the KEGG analysis are provided in the **Supplementary Materials** (**Supplementary Table S8**).

The analysis of Protein-protein interaction (PPI) networks for ACLF-NRGs was conducted using the Cytoscape STRING database, resulting in the construction of a PPI network. LogFC of each gene was obtained from differential analysis to enrich the network graph information (**Figure 4D**).

### Identification and exploration of diagnostic biomarkers

A Random Forest (RF) analysis was conducted on 14 intersecting genes utilizing the Random Forest package. The optimal number of classification trees (**Supplementary Figure 2A**) and the gene importance ranking (**Supplementary Figure 2B**) were obtained. The selection criteria for this analysis were to include genes with mean decrease accuracy that is not zero. The scores of all 14 genes were not zero, so they were all included in the subsequent analysis.

To eliminate the recursive features of the obtained differential genes, the SVM algorithm in R package e1071was employed. RF method was used to sort and calculate the above feature genes, obtaining their significance and ranking, along with the accuracy of each combination of iterations and error rate. The combination with the lowest error rate was selected as the optimal set, and the corresponding genes were designated as feature genes. As shown in the figure below, the highest accurate subset (genes included in the blue dots) of GSE142255 contained four feature genes: FCAR, MMP9, S100A12, CCL5 (**Supplementary Figure 2C**).

**Figure 5:**
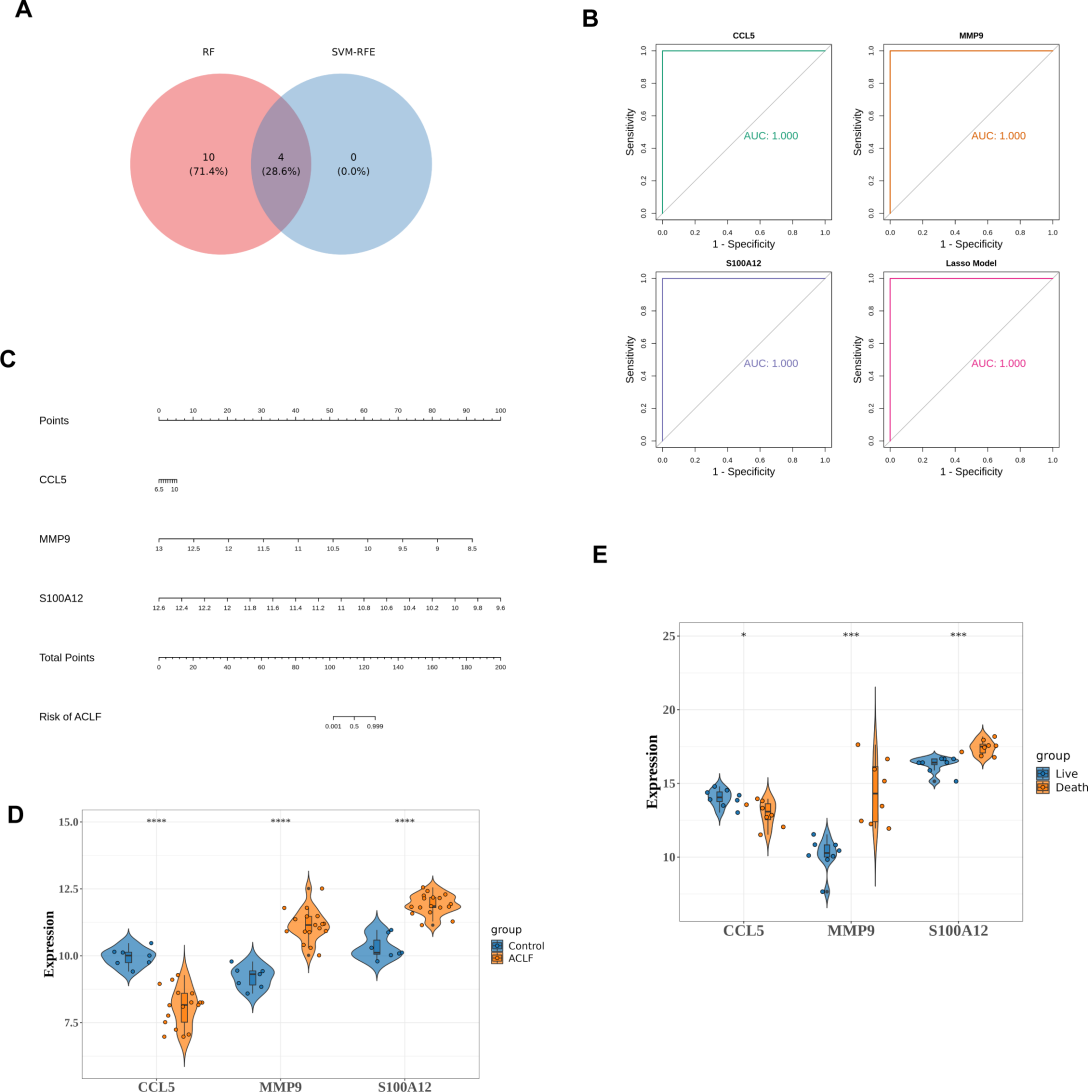
Identifying and validating the diagnostic potential of diagnostic genes. **(A)** Venn diagram to identify key genes. There were four key genes identified as intersections, including FCAR, MMP9, S100A12, and CCL5. **(B)** ROC curves for MMP9, S100A12 and CCL5. **(C)** The nomogram constructed by diagnostic genes. On a point scale, each variable was scored. The total score was determined by summing the individual scores and mapping the total to a lower point scale. Consequently, this allowed for the estimation of the probability of diagnosis. **(D)** Differential expression of three diagnostic genes between the HC and ACLF in training set. ****p<0.0001. **(E)** Differential expressions of diagnostic genes between the death and the survival among ACLF group. *p<0.05, ***p<0.001.

The intersection of genes obtained by RF and SVM-RFE algorithms was used to determine feature genes, as shown in the **Figure 5A**. A total of four biomarkers were obtained, namely FCAR, MMP9, S100A12, and CCL5 (**Figure 5A**).

Subsequent Lasso analysis on these four genes identified three diagnostic genes: MMP9, S100A12, and CCL5. Using the training set data GSE142255, these three diagnostic genes were employed to construct diagnostic models for the ACLF and Control groups, formulated as follows: CCL5 * 1.86 + MMP9 * (-3.21) + S100A12 * (-1.06). This model was then employed to assess the diagnostic capability of the identified genes for ACLF. The Receiver Operating Characteristic (ROC) analysis was conducted on this model, yielding an Area Under the Curve (AUC) value of 1.0, which signifies perfect predictive accuracy and suggests its potential utility in disease diagnosis (**Figure 5B**).

A nomogram was subsequently developed using the diagnostic genes, facilitated by the RMS package (**Figure 5C**). The differential expression levels of MMP9, S100A12, and CCL5 between ACLF and HC are presented in **Figure 5D**, with significant *p*-values less than 0.0001. Among the three diagnostic genes, MMP9 and S100A12 were up-regulated, while CCL5 was down-regulated in ACLF. Furthermore, in GSE168049 dataset, compared to the survival group, S100A12 and MMP9 were up-regulated while CCL5 was down-regulated in the death group, indicating the three diagnostic genes were potential prognostic factors (**Figure 5E**).

### Validation of diagnostic model

**Figure 6:**
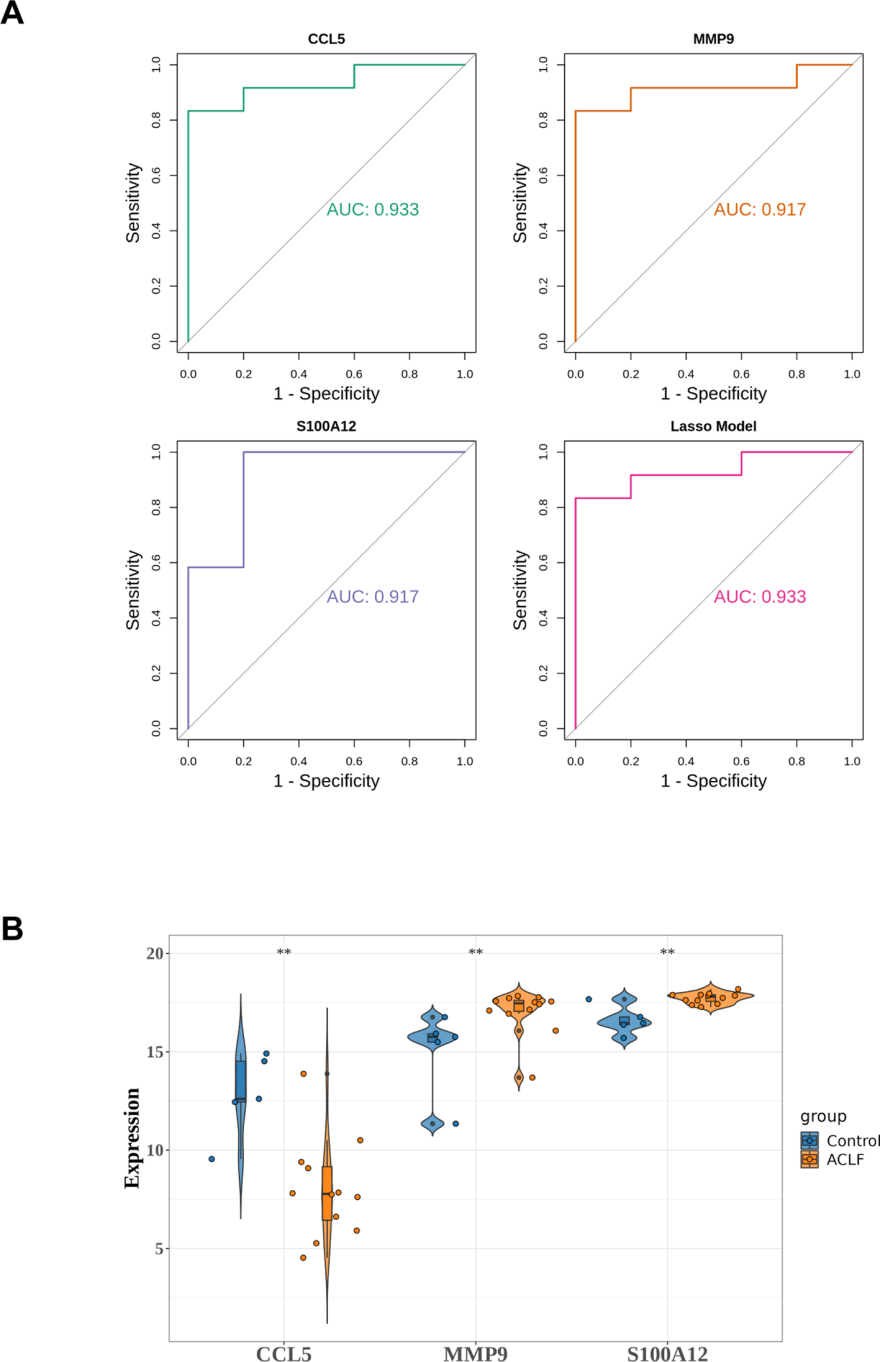
Validation of diagnostic model in verifying dataset. **(A)** ROC analysis of diagnostic genes and LASSO diagnostic model. **(B)** The expression of diagnostic genes in the verifying dataset. **p<0.01.

The lasso diagnostic model constructed by three diagnostic genes was validated using datasets GSE156382, referred to as verifying dataset. In the verifying dataset, the results of the ROC analysis are shown in **Figure 6A**. The model achieved an AUC value greater than 0.9, demonstrating high diagnostic accuracy. Furthermore, the expression levels of the three diagnostic genes were consistent with those in the training set (**Figure 6B**).

### IPA analysis and GSE analysis of diagnostic genes

**Figure 7:**
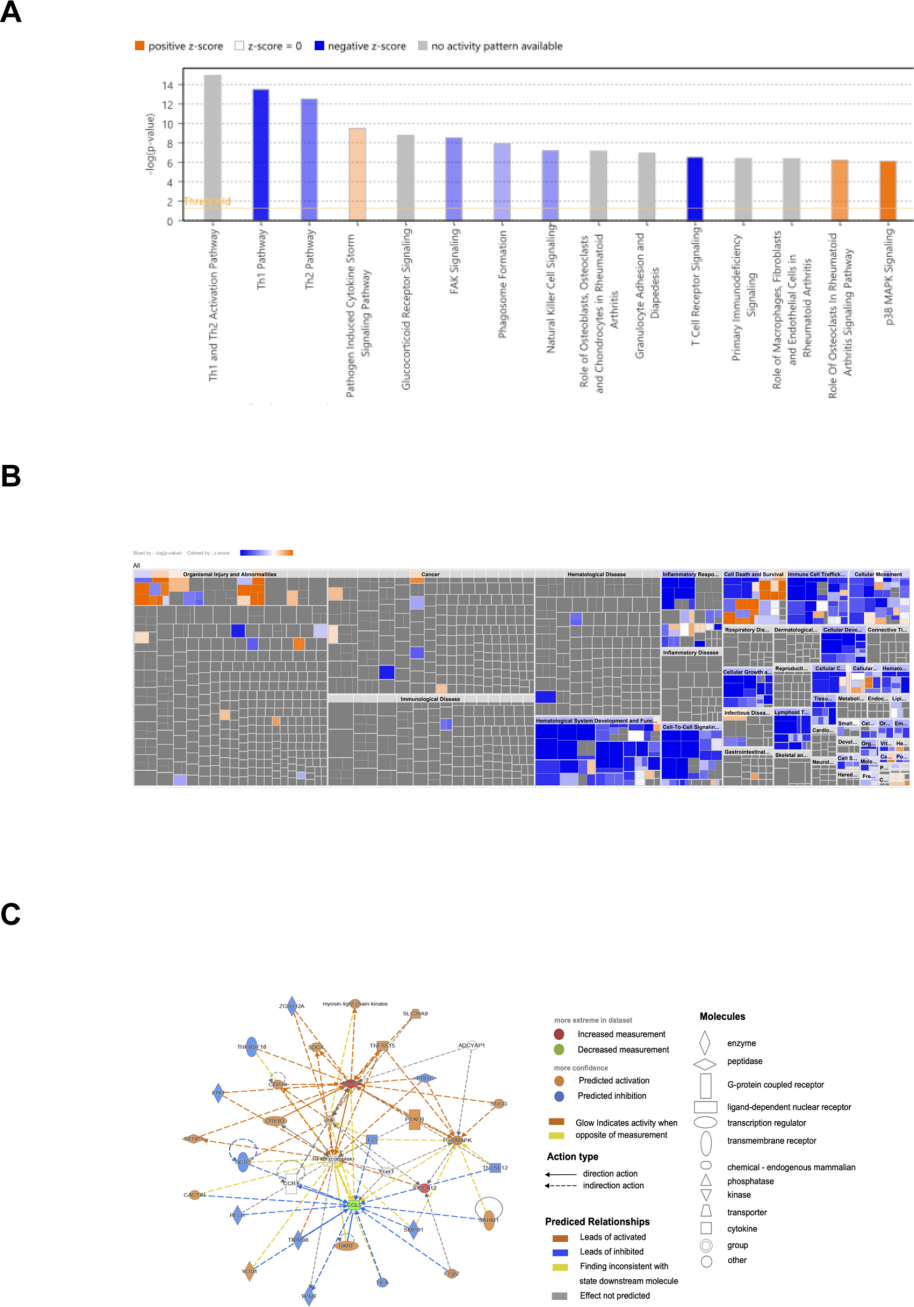
IPA analysis of diagnostic genes. **(A)** Biological functional analysis of diagnostic genes. p<0.05, Z score>2 represented activation, Z score<=2 indicated inhibition; the gray indicated pathways not activated or inhibited, the blue indicated pathways inhibited, the orange indicated pathways activated. **(B)** IPA heatmap for biological functional analysis of diagnostic genes. **(C)** Upstream regulatory factors and downstream target genes of the three diagnostic genes.

In order to further understand the role of diagnostic genes play in the molecular mechanisms of ACLF, IPA was employed to identify the signaling pathways significantly influenced by these genes. The top 15 pathways involved in diagnostic genes were displayed (**Figure 7A**), including four disease-related pathways: pathogen induced cytokine storm, glucocorticoid receptor signaling, natural killer cell signaling and P38 MAPK pathway (19–21). The biological function heatmap enriched by diagnostic genes is presented in **Figure 7B**. Moreover, the upstream regulatory factors and downstream target genes of the three diagnostic genes are presented in **Figure 7C**.

“c2. cp. kegg. v11.0. symbols” were downloaded as the internal reference gene set from MSigDB database. Gene Set Enrichment Analysis (GSEA) were performed on three specific genes: MMP9, S100A12, and CCL5. The KEGG pathways with the top three positive and negative Normalized Enrichment Scores (NES) were displayed (**Supplementary Figure 3**).

### Diagnostic genes correlated with ferroptosis and cuprotosis

**Figure 8:**
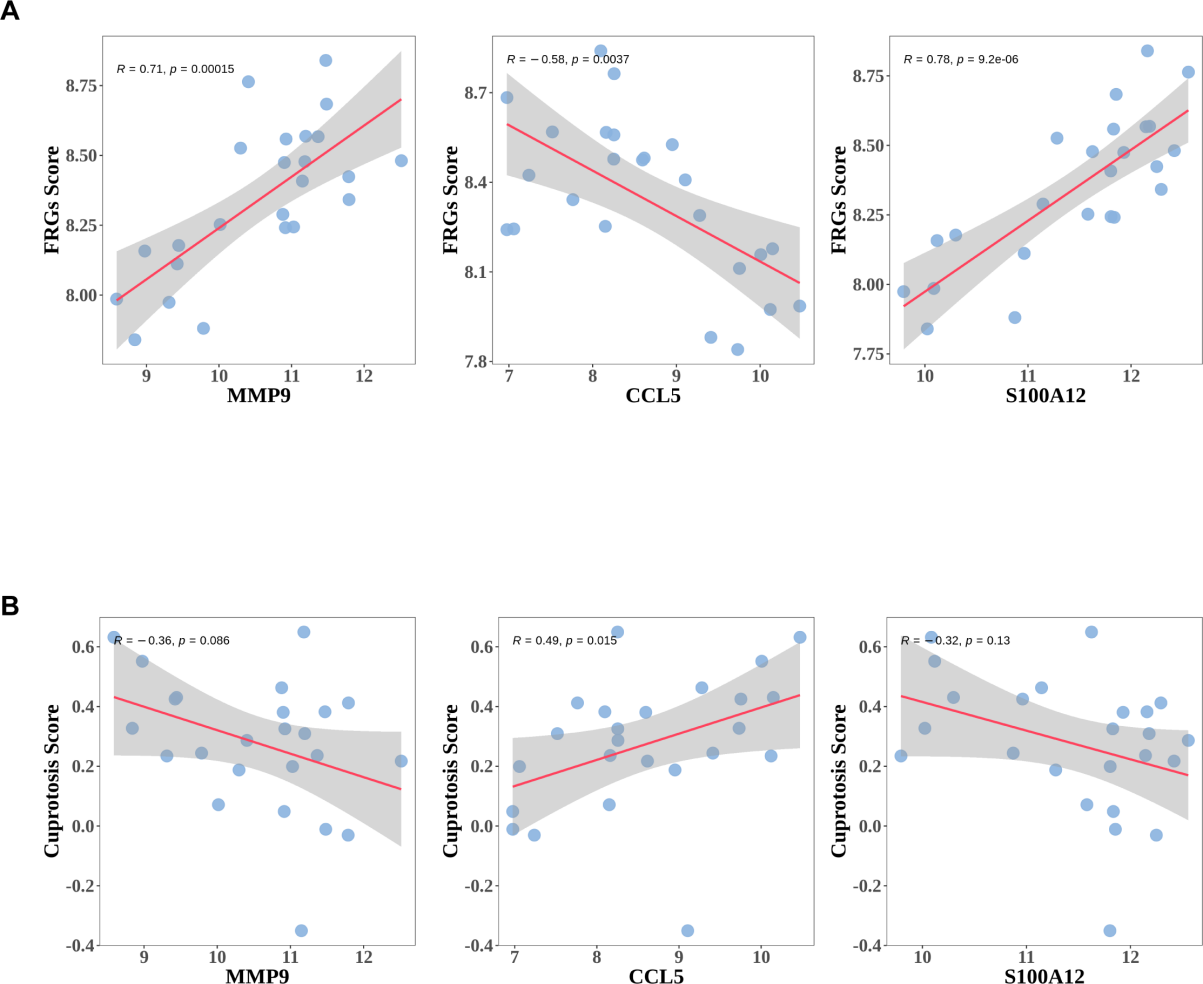
The expressions of diagnostic genes correlated with ferroptosis or Cuprotosis Score. **(A)** The association between expressions of diagnostic genes and ferroptosis ssGSEA score (FRGs Score). **(B)** The association between expressions of diagnostic genes and Cuprotosis ssGSEA score (Cuprotosis Score).

Livers from ACLF patients exhibited key features of ferroptosis, implicating its role in the pathogenesis of ACLF (22). To investigate the correlation between Ferroptosis or cuprotosis and the three diagnostic genes, Pearson correlation analysis was performed. Through published literature, ferroptosis-related genes (FRGs) and cuprotosis-related genes (Cuprotosis) were obtained. Single-sample Gene Set Enrichment Analysis (ssGSEA) was employed to evaluate the ferroptosis ssGSEA score (FRGs Score) and the cuprotosis ssGSEA score (Cuprotosis Score) for the ferroptosis-related and cuprotosis-related gene sets in the GSE142255 dataset. Among the three diagnostic genes analyzed, CCL5 exhibited a significant negative correlation with the FRGs Score, whereas MMP9 and SA1002 demonstrated significant positive correlations with the same score (**Figure 8A**). Conversely, the correlation patterns between these three diagnostic genes and the Cuprotosis Score were found to be opposite to those observed with the FRGs Score (**Figure 8B**). Additionally, three diagnostic genes demonstrated a stronger association with the FRGs Score compared to the Cuprotosis Score.

### Validation of the expression of potential key biomarkers

**Figure 9:**
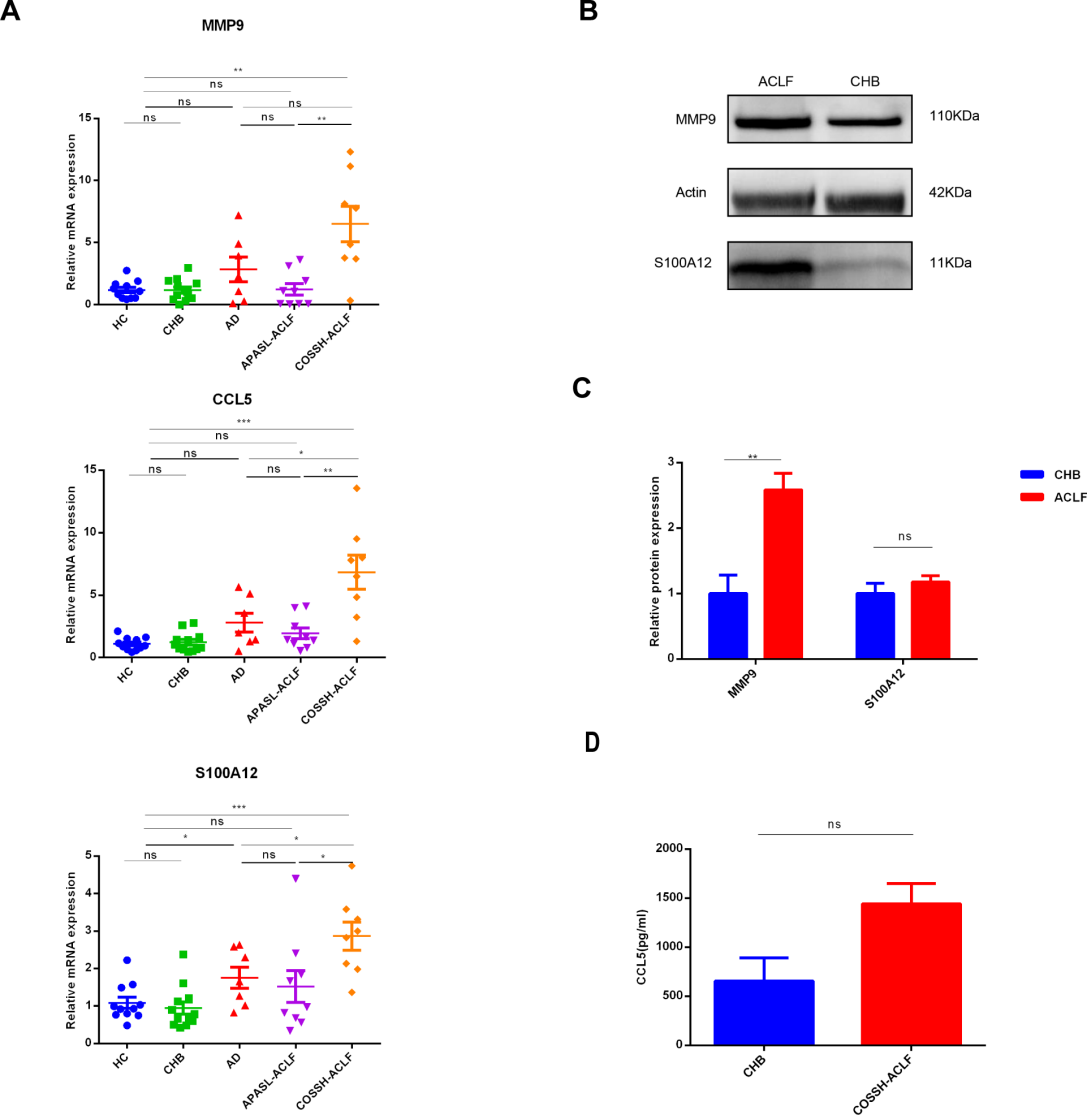
Validation of expression levels of three key biomarkers among the different groups. **(A)** RT−qPCR of expression levels of three key biomarkers among the four groups. RT−qPCR analysis was conducted on peripheral blood white blood cells from 11 HC, 12CHB, 7AD, 9 APASL-ACLF and 8 COSSH-ACLF. **(B, C)** western-blot of expression levels of MMP9 and S100A12 between the CHB and ACLF. western-blot analysis was conducted on peripheral blood white blood cells from 3 CHB and 4 HB-ACLF. **(D)** Levels of plasma CCL5 between the CHB and COSSH-ACLF. ELISA analysis was conducted on plasma from 11 CHB and 62 HB-ACLF. HC healthy control, CHB chronic hepatitis B, HB-ACLF HBV-related acute-on-chronic liver failure, APASL Asian-Pacific Association for the Study of the Liver, COSSH Chinese Group on the Study of Severe Hepatitis B, *p < 0.05, **p < 0.01, ***p<0.001, ns, not statistically significant.

qRT-PCR, longside WB or ELISA was performed on peripheral white blood cells or plasma samples from HC, CHB and ACLF to validate the expression levels of the three diagnostic genes. A novel diagnostic criterion for HB-ACLF was established, known as the Chinese Group on the Study of Severe Hepatitis B-ACLF (COSSH-ACLF) (23). According to this criterion, patients with CHB exhibiting a total bilirubin (TB) level of ≥12 mg/dL and an international normalized ratio (INR) of ≥1.5 should be diagnosed with ACLF, irrespective of the presence of cirrhosis. This criterion includes patients with higher TB levels than those defined by APASL. Since all the patients included were HB-ACLF except for one case, the qRT-PCR ACLF cohort was further stratified into two groups: patients with TB ≥12 mg/dL were categorized under the COSSH-ACLF group, while those with TB <12 mg/dL were classified under the APASL-ACLF group. The mRNA expression levels of the three diagnostic genes were significantly elevated in the COSSH-ACLF group compared to the HC, CHB and APASL-ACLF groups (**Figure 9A**). Additionally, there were an increase in S100A12 and CCL5 expression in COSSH-ACLF than AD. Among the three key genes, only the expression of S100A12 were upregulated in AD, compared with HC and CHB. No statistically significant differences were observed among the HC, CHB and APASL-ACLF groups (**Figure 9A**). However, at the protein level, only MMP9 was confirmed to be more highly expressed in the ACLF group compared to the CHB group (**Figures 9B, C**). No significant differences were found between the CHB and ACLF groups in the protein expression of S100A12 and CCL5 (**Figures 9B–D**).

### Enzyme-linked immunosorbent assay of MMP9

**Figure 10:**
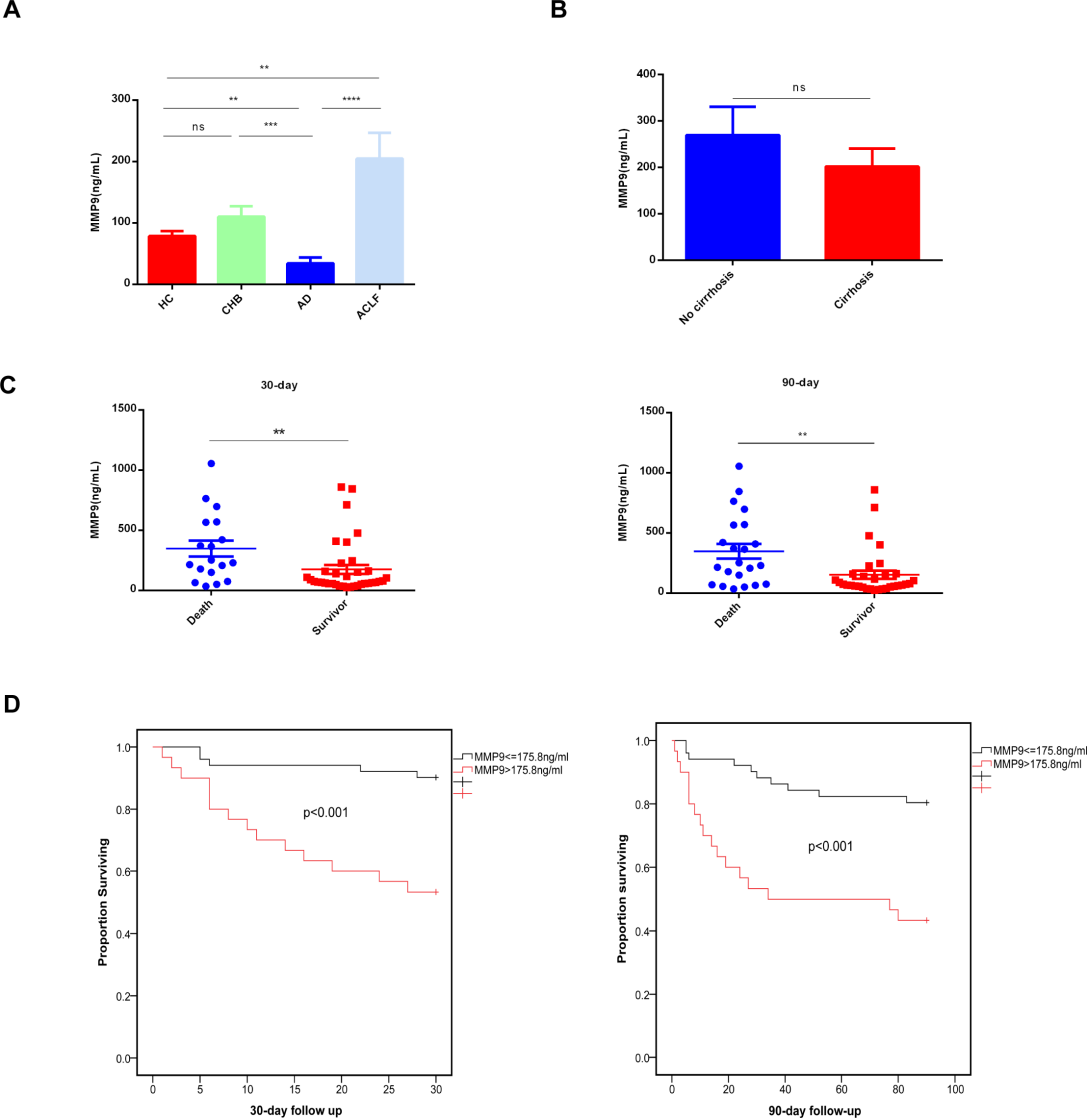
Concentrations of plasma MMP9 correlated with the outcome of ACLF patients. **(A)** Concentrations of Plasma MMP9 in the different groups. ELISA analysis was conducted on plasma samples from 15HC, 11CHB, 8AD, 40ACLF. HC healthy control, CHB chronic hepatitis B, AD acute decompensated cirrhosis, ACLF acute-on-chronic liver failure. (**p<0.01, ***p<0.001, ****p<0.0001, ns, not statistically significant). **(B)** Concentrations of Plasma MMP9 in the cirrhosis and non-cirrhosis. ELISA analysis was conducted on plasma samples from cirrhosis (n=30) and non-cirrhosis (n=24) (ns, not statistically significant). **(C)** Concentrations of Plasma MMP9 among the death and the survival. (**p<0.001) **(D)** Comparison of K-M survival curves between 81 ACLF patients with high or low plasma MMP9. A log-rank test was used to compare 30-day and 90-day cumulative survival between groups.

Among the three key genes, MMP9 expression exhibited the most significant difference between the death and survival groups (**Figure 5E**). Consequently, additional ELISA analyses were conducted to assess MMP9 levels. Specifically, MMP9 ELISA was conducted on plasma samples from 15 HC, 11 patients with CHB, 8 patients with HB-AD, and 40 patients with HB-ACLF to validate the differential expression levels of MMP9. The results indicated a significant elevation of MMP9 levels in the ACLF group compared to the other three groups (**Figure 10A**). MMP9, also known as type IV collagenase, is implicated in fibrosis. There was no significant difference in plasma MMP9 levels between patients with cirrhosis and those without (**Figure 10B**). It was interesting to note that plasma MMP9 levels in AD patients were significantly lower than those in HC and CHB patients. CHB and HC did not differ significantly in MMP9 levels (**Figure 10A**). Further ELISA analysis on additional cases of ACLF revealed that plasma MMP9 levels were significantly higher in patients who succumbed to the condition compared to those who survived (**Figure 10C**). ACLF patients with elevated MMP9 levels (>175.8 ng/ml) exhibited higher short-term mortality rates within both 30 and 90 days (**Figure 10D**, p<0.001).

### Drug prediction and construction of kinase-TF-mRNA-miRNA network

**Figure 11:**
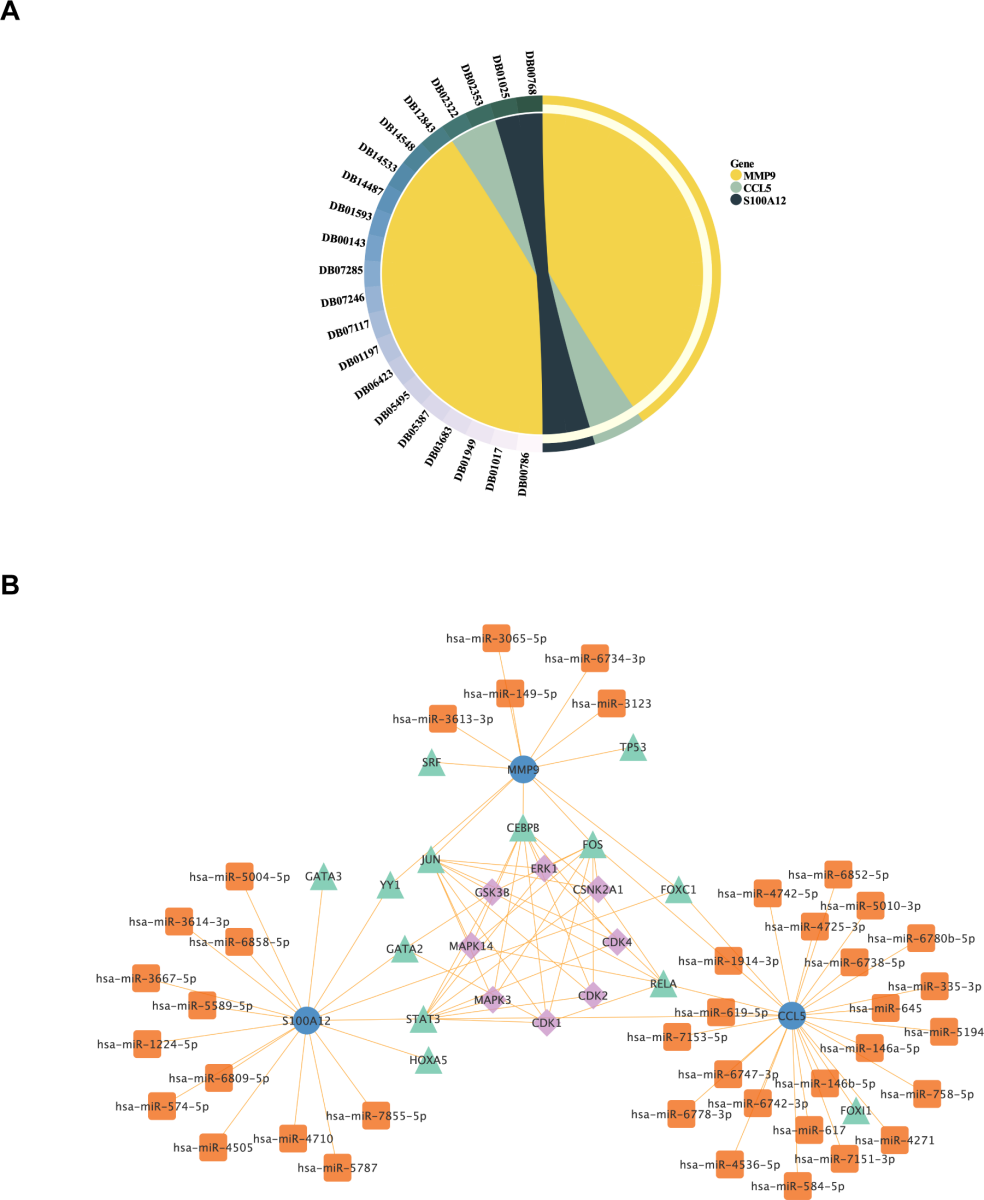
Analysis of Potential Therapeutic Targets and Regulatory Networks for ACLF. **(A)** Small molecule drug prediction for diagnostic genes had been conducted. The symbols on the left side represented the drug’s ID in the DrugBank database, while the right side represented the 3 diagnostic genes that were the focus of the analysis. **(B)** The construction of Kinase-TF-mRNA-miRNA network. Blue dots represented diagnostic genes, green triangles represented transcription factors, orange squares represented miRNAs, and purple diamonds represented Kinase.

Drug Bank database was used to predict small molecule drugs for diagnostic genes. A total of 21 drugs were obtained targeting the three diagnostic genes. Specifically, CCL5 and S100A12 each yielded two predictive results, while MMP9 yielded 17 predictive results (**Figure 11A**). Among the 17 predictive drugs identified for targeting MMP9, the following were approved: Minocycline, Captopril, Glutathione, Zinc, Zinc acetate, Zinc chloride, and Zinc sulfate. Additionally, Amlexanox and Olopatadine were the two approved predictive drugs targeting S100A12. No approved drugs were identified for CCL5; however, two experimental drugs, Heparin Disaccharide I-S and Heparin Disaccharide III-S, were noted. Detailed information regarding these drugs was provided in the **Supplementary Materials** (**Supplementary Table S9**). The approved drugs would be discussed below.

Transcription factors (TF) that regulate diagnostic genes were retrieved from NetworkAnalyst database (https://www.networkanalyst.ca/). Then the upstream kinases of TF were predicted by X2Kgui and miRNA of diagnostic genes were predicted by using miRDB database. At last, The Kinase-TF-mRNA-miRNA network was constructed by Cytoscape software (**Figure 11B**).

The prediction of these drugs aims to screen for drugs that may affect the pathological process of ACLF by regulating the expression or function of these genes. Through drug screening and regulatory network analysis, we provided preliminary insights into potential treatment strategies and targets. Moreover, The Kinase-TF-mRNA-miRNA network also provided the potential regulatory mechanisms of these genes, establishing a basis for mechanistic research on these key genes in ACLF.

## Discussion

In the present study, we identified neutrophil-related genes in ACLF patients by WGCNA algorithms. Our study concentrated on 14 genes identified at the intersection of NEGs, DEGs, and ModelGenes. This strategic selection is intended to precisely elucidate the specific role of neutrophils in the pathological processes of ACLF. By focusing on these particular genes instead of the entire set of DEGs for pathway analysis, we aim to achieve a more detailed and specific understanding of the fundamental impact of neutrophils on the inflammatory mechanisms of ACLF. This approach minimizes the risk of obtaining non-specific results that can occur with broader analyses, thereby effectively preserving the clarity of key molecular interactions. In our study, MMP9, S100A12 and CCL5 were found to be three diagnostic and prognostic genes in ACLF. The expression levels of the three key genes were found to be correlated with both ferroptosis and cuprotosis. Furthermore, potential drugs and regulatory mechanisms targeting these diagnostic genes were predicted using the DrugBank database and Cytoscape software, respectively. These findings suggest novel biomarkers for the diagnosis and prognosis of ACLF, thereby offering research targets for future exploration of molecular mechanisms and drug design. However, further research is required to validate these results and to elucidate the underlying mechanisms.

MMP9, S100A12 and CCL5 were identified as the top three diagnostic genes. IPA analysis revealed that the key genes are predominantly involved in the pathogen induced cytokine storm, glucocorticoid receptor signaling, which are closely related to the disease. While the pathogenesis of HB-ACLF is still unclear, growing evidence indicates sustained systemic inflammatory responses induced by cytokine storm are crucial to multiple organ failure and high mortality (24, 25). There has been a growing number of studies showing the therapeutic efficacy of glucocorticoids in the management of HB-ACLF. A recent systematic review demonstrated that patients with HB-ACLF who receive glucocorticoid (GC) therapy exhibit lower total bilirubin (T-bili) levels, resulting in a significant reduction in in-hospital mortality and ascites events (26). Short-term treatment with low doses of GC, in combination with nucleoside analogues during the early stages of HB-ACLF, has been shown to be both safe and effective (27). Additionally, a recent study introduced the HITAS score for identifying patients with HB-ACLF who are likely to respond favorably to glucocorticoid treatment (28). However, glucocorticoids are associated with numerous adverse effects, such as gastrointestinal bleeding and infections. Consequently, there is a pressing need to identify novel therapeutic targets.

Our study firstly discovered plasma MMP9 levels were higher in patients with HB-ACLF compared to healthy controls, individuals with CHB, and those with HB-AD. MMP9 levels in plasma and MMP9 mRNA expression in neutrophils were both associated with poor outcomes of ACLF, indicating MMP9 may play a pathogenic role, which worth further study. Previous study has highlighted a intriguing phenomenon: reducing hypoxic tumor environments may decrease neutrophil recruitment, but neutrophils are extraordinarily effective at killing cells. These activated neutrophils secrete ROS and MMP-9, leading to epithelial basement membrane degradation (29). Our previous research has shown that patients with ACLF had more activated neutrophils than those with chronic hepatitis B, releasing more ROS. While stimulated with bacterials, neutrophils from patients with ACLF would be overwhelmingly activated, releasing much more ROS than CHB and HC (5). Consequently, we hypothesize that the activated neutrophils in ACLF may contribute to the degradation of various basement membranes and the following development of multiple organ disorders through the release of ROS and matrix metalloproteinase-9 (MMP9). Additionally, MMP9 has recently been identified as a biomarker for M1 macrophages, which are implicated in the immune and inflammatory responses associated with the progression of Rheumatoid Arthritis. This finding is further supported by evidence indicating that the inhibition of MMP9 protein expression exhibits anti-inflammatory effects in lipopolysaccharide stimulated RAW264 cell lines (30). Thus, MMP9 might also play a pro-inflammatory role in ACLF.

CCL5 might be a potent predictor for ACLF. Its expression is strongly expressed in the liver samples of patients with HB-ACLF (31). Circulating monocytes from ACLF death group expressed significantly higher chemokine levels, including CCL5 (32). Consistent with those results, in our qRT-PCR analysis, we observed elevated expression levels of CCL5 mRNA in patients with ACLF compared to those with CHB and HC. Contrarily, data from both the training and validation cohorts obtained from the GEO showed inverse trends. This discrepancy may be attributed to the underlying etiology of ACLF. Specifically, in the GEO cohorts, approximately 88% to 100% of ACLF cases were non-viral in origin, whereas our qRT-PCR study exclusively involved patients with HB-ACLF. It is important to note that the roles of various chemokines and their receptors differ significantly in the pathogenesis of distinct liver diseases. In the progression of chronic viral hepatitis, CCL5 and CXCL10 modulate the cytopathic and antiviral immune responses of natural killer cells and T lymphocytes. Conversely, in the development of nonalcoholic steatohepatitis, there is an upregulation of CCL2 and its receptor in the liver, facilitating macrophage recruitment, inflammation, steatosis and fibrosis, as well as in adipose tissue (33). Nevertheless, in our study, serum CCL5 levels did not differ significantly between the groups, which contrasts with findings from a previous study that reported a marked increase in serum CCL5 levels in CHB patients as the liver inflammation progressed from mild to moderate-to-severe stages (34). However, this previous study also indicated an opposite trend in patients with HBV-related cirrhosis (34), suggesting that the presence or absence of cirrhosis could substantially influence the results. As our study did not differentiate between patients with and without cirrhosis, this factor may account for the discrepancies observed between our findings and those of the prior study. In alignment with the GEO data, our study demonstrated that mRNA expression of S100A12 in circulating peripheral white blood cells was elevated in patients with COSSH-ACLF compared to those with HC, CHB, AD, and APASL-ACLF. However, protein levels of S100A12 did not differ between COSSH-ACLF and CHB, which contrasts with previous findings indicating that patients with HB-ACLF exhibited significantly higher serum levels of S100A12 compared to those with cirrhosis, CHB, and HC (35). This discrepancy may be attributed to two factors. First, the CHB patients in our study were in the active phase of hepatitis, characterized by marked elevated transaminases, unlike those in the previous report. Second, the limited sample size of our study may have affected the results. Further studies with larger cohorts are necessary to elucidate the diagnostic and prognostic value of S100A12 in ACLF.

As an iron-dependent necrosis with non-apoptotic properties, ferroptosis is induced by the interaction of iron ions with ROS, resulting in lipid peroxidation and Fe2+ accumulation (36). The liver owns a considerable amount of iron and various oxidases, as is universally acknowledged. Hepatic cells exposed to high levels of iron undergo severe oxidative stress, increasing the production of ROS and lipid peroxidation. As a result, iron metabolism pathways are disrupted, ultimately leading to ferroptosis (37), which contributes to a variety of liver diseases (38, 39). It has been demonstrated that the development of ACLF is related to disruptions in iron metabolism, imbalances in amino acid antioxidant systems, and the occurrence of lipid peroxidation (36). Inhibition of ferroptosis alleviate liver inflammation in ACLF, indicating ferroptosis plays an important role in the pathogenesis of ACLF (22, 36, 40). Investigations suggested hemin-induced macrophage ferroptosis promoted MMP2/9 overexpression (41). CCL5 might be the key factor in regulating ferroptosis after intracerebral hemorrhage (42). In our study, the three key genes exhibited a strong association with ferroptosis, suggesting that the over-expression of MMP9 in ACLF may be a consequence of ferroptosis. Furthermore, CCL5 may influence the progression of ACLF by modulating ferroptosis. There has been no studies focusing on the relationship between S100A12 and ferroptosis. These findings offer a basis for future investigations into the distinct roles of copper-induced cell death and iron-induced cell death in the pathogenesis of this disease.

This study has identified a selection of pharmacological agents to explore their potential efficacy in the treatment of ACLF. Specifically, drugs targeting MMP9, including minocycline, captopril and glutathione, have demonstrated inhibitory effects on MMP9, a critical enzyme in the inflammatory process (43). Minocycline exerts anti-inflammatory effects on monocytes, neutrophils, microglial cells, and neurons. Minocycline mitigates neutrophil-related tissue injury by inhibiting neutrophil migration and degranulation, as well as suppressing the formation of oxygen radicals. Additionally, it inhibits the inducible form of nitric oxide synthase (iNOS), Interleukin-1beta-Converting enzyme (ICE-1) and lipopolysaccharide-induced mRNA which contribute to inflammation (44, 45). Captopril and glutathione have been documented to exhibit significant free radical scavenging properties (46–49).

Pharmacological agents targeting the S100A12, including Amlexanox and TBK1. Amlexanox has been shown to down-regulate the immune system and attenuate downstream TBK1 signaling (50). Olopatadine is an antiallergic agent that functions as a selective histamine H1 receptor antagonist, demonstrating inhibitory effects on the release of inflammatory lipid mediators, such as leukotriene and thromboxane, from human polymorphonuclear leukocytes and eosinophils (51).

By inhibiting MMP9 or S100A12 activity, these agents are anticipated to mitigate neutrophil activation, migration and tissue damage, thereby offering a promising therapeutic strategy for patients with ACLF.

This study elucidated the regulatory interactions between multiple microRNAs (miRNAs) and key genes, including CCL5, MMP9, and S100A12, through comprehensive miRNA analysis. Notably, hsa-miR-146a-5p and hsa-miR-5010-3p were identified as dysregulated in ACLF and were associated with patient prognosis (52, 53). Previous studies have indicated that miRNAs are involved in the regulation of NET formation and neutrophil apoptosis during inflammatory processes (54, 55). Consequently, these miRNAs may also play significant roles in modulating the inflammatory response and neutrophil function in ACLF. Future research should aim to further elucidate and validate the specific functions and mechanisms of action of these miRNAs in ACLF, while also investigating their potential as therapeutic agents.

While this study effectively investigated gene expression patterns associated with neutrophils in ACLF, offering novel insights for disease diagnosis and therapeutic targets, we acknowledge certain limitations inherent in the research. Specifically, the restricted sample size and the necessity to enhance the diversity of patient sources currently constrain the generalizability of the findings. In the future, more samples and multiple centers will be needed to confirm the role of these key genes in diagnosis and prognosis. In addition, the plasma levels of CCL5 showed no difference between ACLF and CHB, indicating circulating CCL5 might not play an important role in ACLF. Through bio-informatics analysis, we identified these key genes, examined their functions and features, and predicted their regulatory networks and potential targeted drugs. However, it is important to note that these findings are preliminary predictions and necessitate further experimental validation.

## Data Availability

The original contributions presented in the study are included in the article/**Supplementary Material**. Further inquiries can be directed to the corresponding author.
