# Supplementary material for: Identification and validation of neutrophil-related biomarkers in acute-on-chronic liver failure
Source: Front Immunol. 2025 Feb 25;16:1477342. doi: 10.3389/fimmu.2025.1477342 (PMC11893565; doi:10.3389/fimmu.2025.1477342)
Supplement: Supplementary file 1 [file DataSheet1.docx]

**

**

**SUPPLEMENTARY FIGURE 1** | Outlier sample detection.


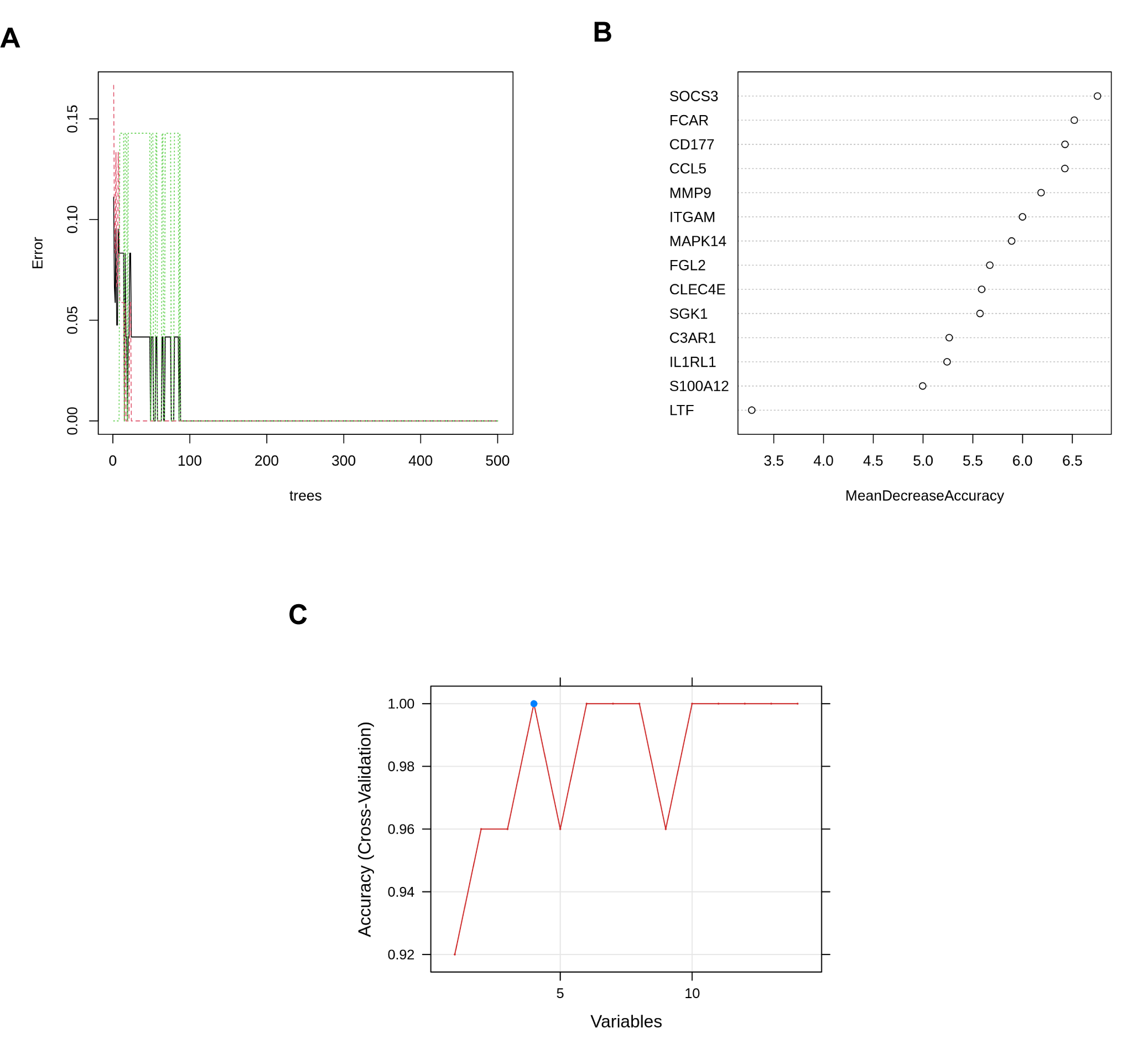


**SUPPLEMENTARY FIGURE 2** | Screening feature genes. (A) Selection of the Number Parameter of Random Forest Decision Trees. The horizontal axis indicated the number of decision trees that make up a random forest, and the vertical axis indicated the corresponding error rate. (B) Identify feature genes. The horizontal axis indicated the order of importance, and the vertical axis indicated the genes sorted by importance. (C) Accuracy curves for different subsets of variables in GSE142255. The horizontal axis represented subsets composed of different numbers of genes, while the vertical axis represented accuracy.

**
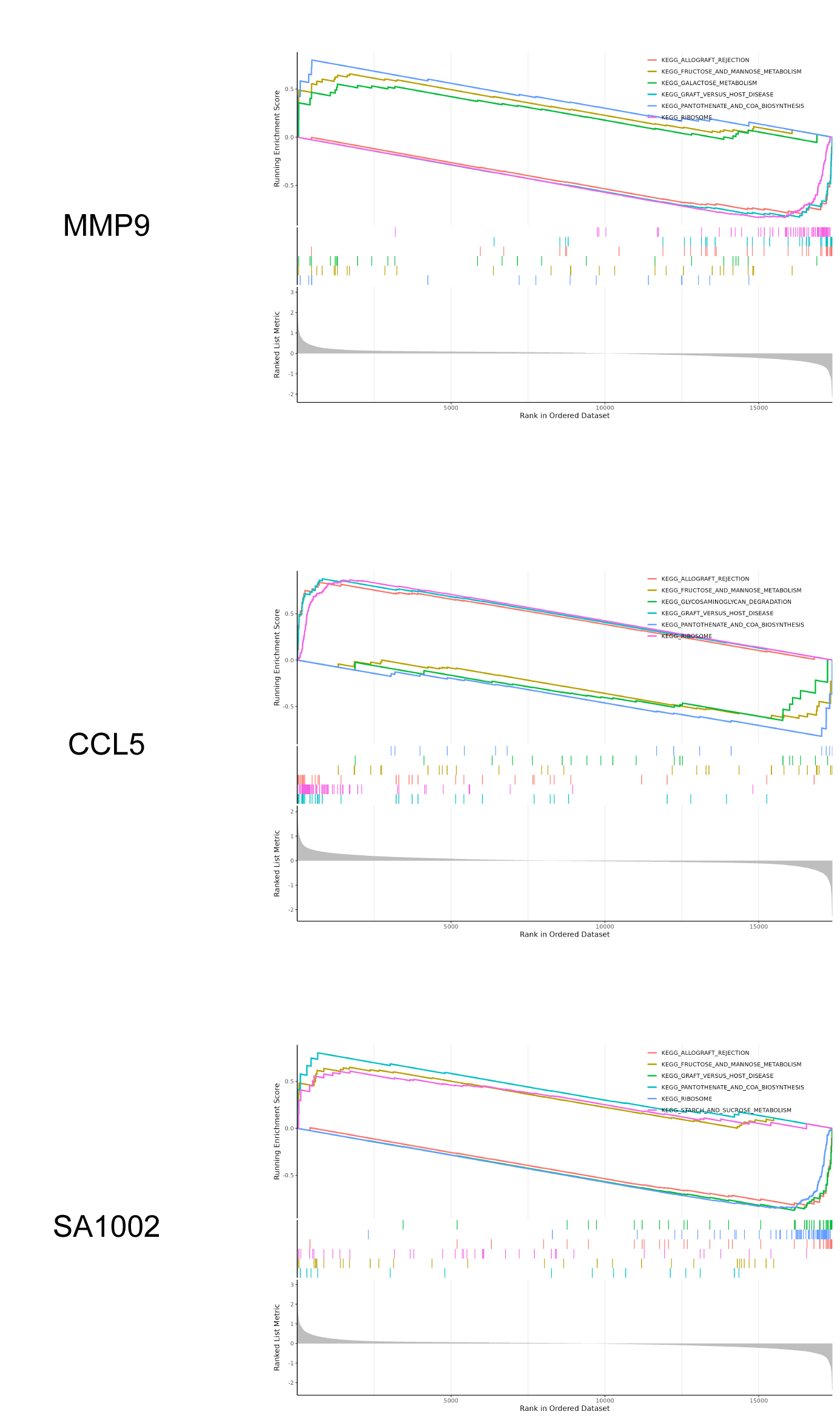
**

**SUPPLEMENTARY FIGURE 3** | KEGG pathway enriched with three diagnostic genes.
