# Supplementary material for: Identification and validation of neutrophil-related biomarkers in acute-on-chronic liver failure
Source: Front Immunol. 2025 Feb 25;16:1477342. doi: 10.3389/fimmu.2025.1477342 (PMC11893565; doi:10.3389/fimmu.2025.1477342)
Supplement: Supplementary file 3 [file Table2.docx]

**Table S2.** **Baseline characteristics of included subjects for RNA and WB.**

|  | ACLF (n=21) | AD (n=7) | CHB (n=15) | HC (n=11) |
| --- | --- | --- | --- | --- |
| Age (years), Median (IQR) | 46.0 (32.0-52.5) | 52.0(45.0-58.0) | 45.0 (41.0-52.0) | 48.0 (33.0-55.0) |
| Male (%) | 15 (71.4) | 6 (85.7) | 11 (73.3) | 8 (72.7) |
| **Clinical feature** |  |  |  |  |
| HBV infection | 20 (95.2) | 7 (100) | 7 (100) | - |
| Ascites (%) | 18 (85.7) | 7 (100) | 0 (0) | - |
| Cirrhosis (%) | 12 (57.1) | 7 (100) | 0 (0) | - |
| UGIB (%) | 0 (0) | 0 (0) | 0 (0) | - |
| HE (%) | 6 (28.6) | 0 (0) | 0 (0) | - |
| **Laboratory data** |  |  |  |  |
| WBC (×10^9^/L), Median (IQR) | 6.6 (4.7-8.8) | 4.1 (2.7-4.4) | 6.2 (5.5 -7.3) | n.a. |
| ALT (IU/L), Median (IQR) | 162 (45.5-1062.5) | 26.0 (12.0-849.0) | 80.0 (24.0 -571.0) | n.a. |
| Albumin (g/L), Median (IQR) | 29.2 (26.5-32.9) | 31.0 (25.6-33.5) | 37.8(32.6 -41.9) | n.a. |
| Bilirubin (mg/dL), Median (IQR) | 11.9 (9.9-20.3) | 4.9 (2.9-10.8) | 1.1(0.8-2.5) | n.a. |
| INR，Median (IQR) | 2.0 (1.8-2.4) | 1.3 (1.2-2.2) | 1.1 (1.0- 1.23) | n.a. |
| Creatinine (mg/dL), Median (IQR) | 0.7 (0.7-0.9) | 0.7 (0.6-0.8) | 0.8 (0.7-0.9) | n.a. |
| **Scores** |  |  |  |  |
| CTP, Mean (±SD) | 11.5 ± 1.6 | 9.9 ± 2.2 | 5.8 ± 1.1 | n.a. |
| MELD, Mean (±SD) | 21.8 ± 3.5 | 14.8 ± 2.1 | 7.1 ± 4.5 | n.a. |

ACLF acute-on-chronic liver failure, AD decompensated cirrhosis, CHB chronic hepatitis B, HC healthy controls, UGIB upper gastrointestinal bleeding, HE hepatic encephalopathy, WBC white blood cell count, INR international normalized ratio, CTP Child—Turcotte-Pugh, MELD Model for End-stage Liver Disease.
